# Supplementary material for: Disordered region of nuclear membrane protein Bqt4 recruits phosphatidic acid to the nuclear envelope to maintain its structural integrity
Source: J Biol Chem. 2024 May 31;300(7):107430. doi: 10.1016/j.jbc.2024.107430 (PMC11253665; doi:10.1016/j.jbc.2024.107430)
Supplement: Supporting information [file mmc3.pdf]

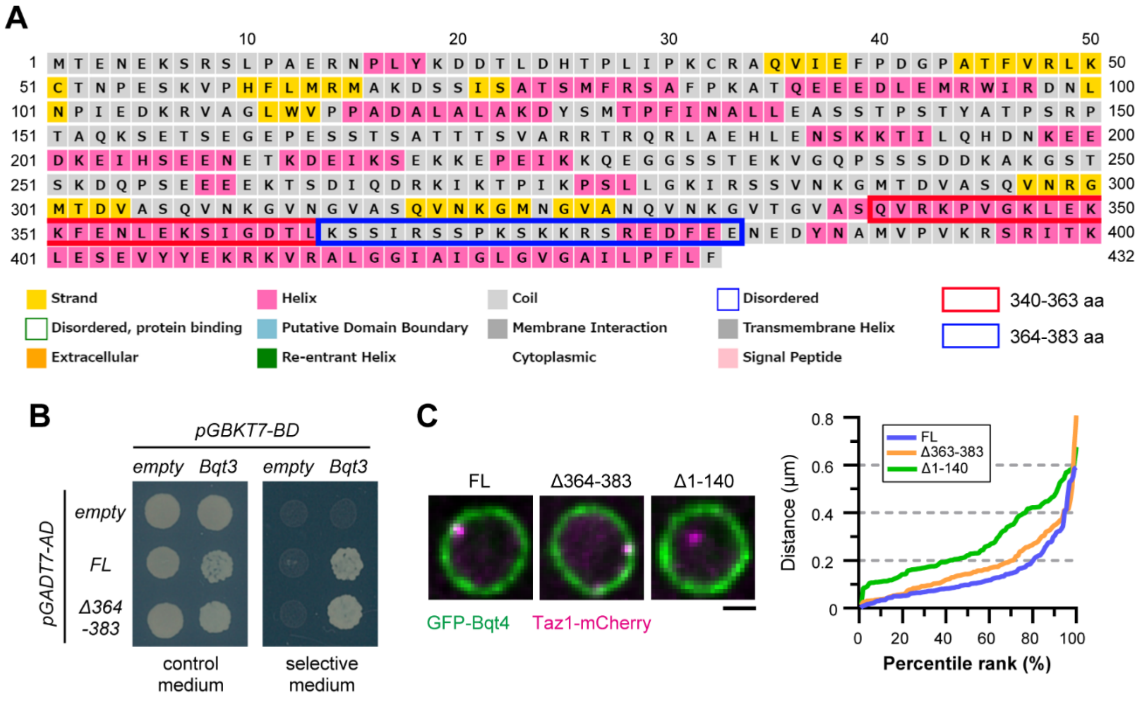

Supplementary Fig. S1. KSE region characteristics

A. Secondary structure prediction of Bqt4 by PSIPRED 4.0. The sequences surrounded by red and blue frames indicate 340-363 aa and 364-383 aa, respectively.

B. Yeast two-hybrid assay. *S. cerevisiae* cells expressing the full-length Bqt4 (FL) and the Bqt4 Δ364-383 fragment with Bqt3 were grown on a medium with different auxotrophy (control and selective medium) for 3 days. Fusion proteins with AD (pGADT7-AD) and BD (pGBKT7-BD) are shown on the left and top, respectively.

C. Distance between the NE and telomeres. (Left) Full-length (FL) and fragments (Δ364-383 and Δ1-140) of Bqt4 tagged with GFP (green) were co-expressed with Taz1-mCherry as a maker for telomere (magenta). (Right) Distance between the NE to telomeres was measured and plotted as a percentile rank. The scale bar represents 1 μm.

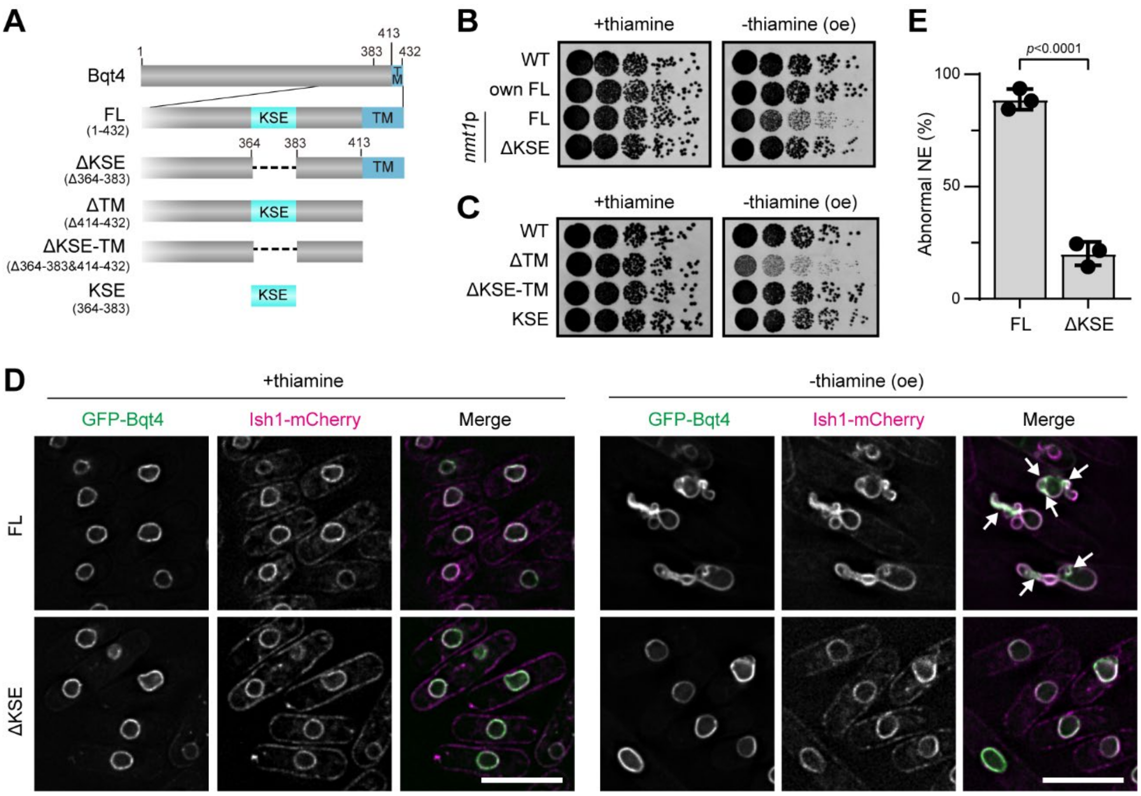

Supplementary Fig. S2. The KSE region is required for cell growth, but insufficient for dominant negative defects

A. Schematic diagrams of KSE fragments. TM denotes the transmembrane domain.

B. Spot assay of the cells overexpressing ΔKSE fragment. GFP-tagged Bqt4 fragments indicated on the left were expressed under authentic (own) or *nmt1* (*nmt1p*) promoters. The growth of the cells was observed as described in Fig. 1A. oe represents overexpression.

C. Spot assay of cells overexpressing the Bqt4 fragments. GFP-Bqt4 fragments indicated on the left were expressed under the *nmt1* promoter. The growth of the cells was observed as described in (B).

D. NE deformation by Bqt4 overexpression. GFP-tagged FL or ΔKSE were expressed with Ish1-mCherry as a nuclear membrane marker (Asakawa et al., 2022) in *S. pombe* cells. The cells were cultured in the EMMG medium with or without thiamine (labeled "+thiamine" and "-thiamine (oe)," respectively), and then observed using fluorescence microscopy. Arrows indicate the region of Bqt4 enrichment. The scale bars represent 5 µm.

E. Quantification of abnormal NE morphology. Percentage of the cells that showed abnormal NE morphology in (D) was quantified from three independent experiments. The mean and standard deviation were shown. The statistical significance was assessed using two-tailed Welch's *t*-test.

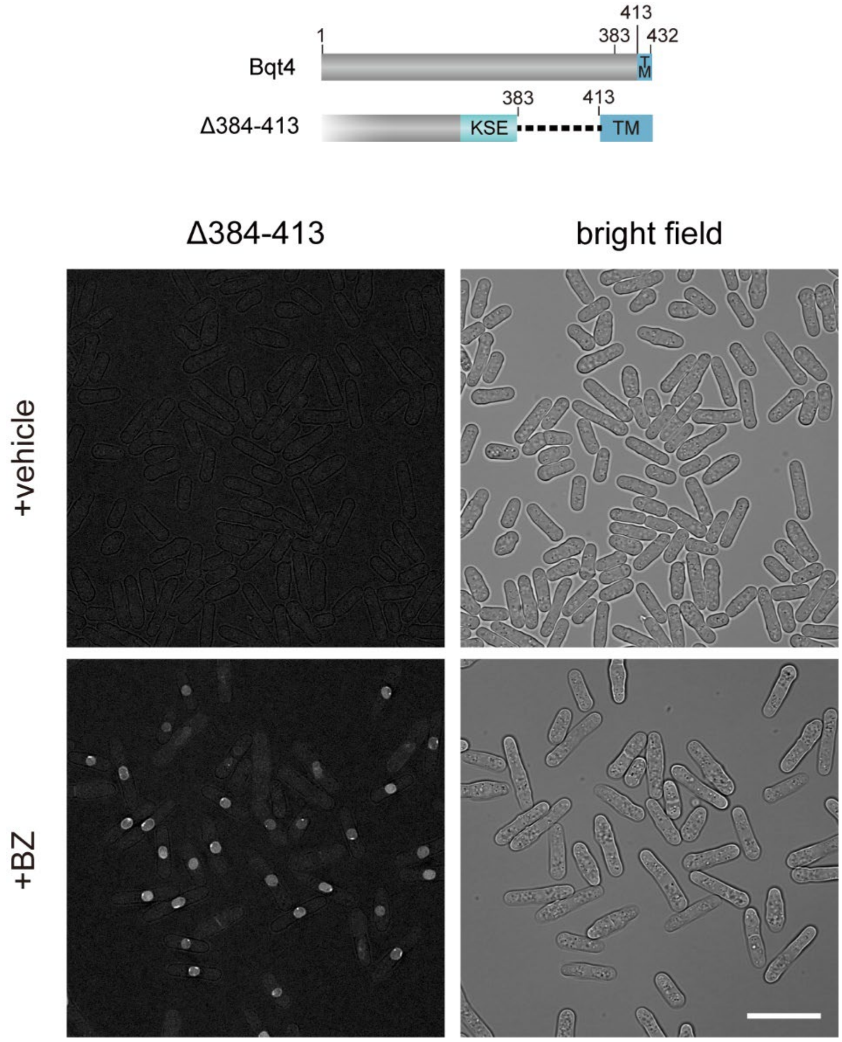

Supplementary Fig. S3. Bqt4Δ384-413 protein fragment undergoes degradation
GFP-tagged Bqt4Δ384-413 fragment was expressed in the *lem2*-shut-off *bqt4*Δ cells and observed in the presence (+BZ) or absence (+vehicle) of 1mM bortezomib, a proteasome inhibitor effective in *Schizosaccharomyces pombe* cells. Bright-field images are shown on the right side. Scale bar represents 20 μm.

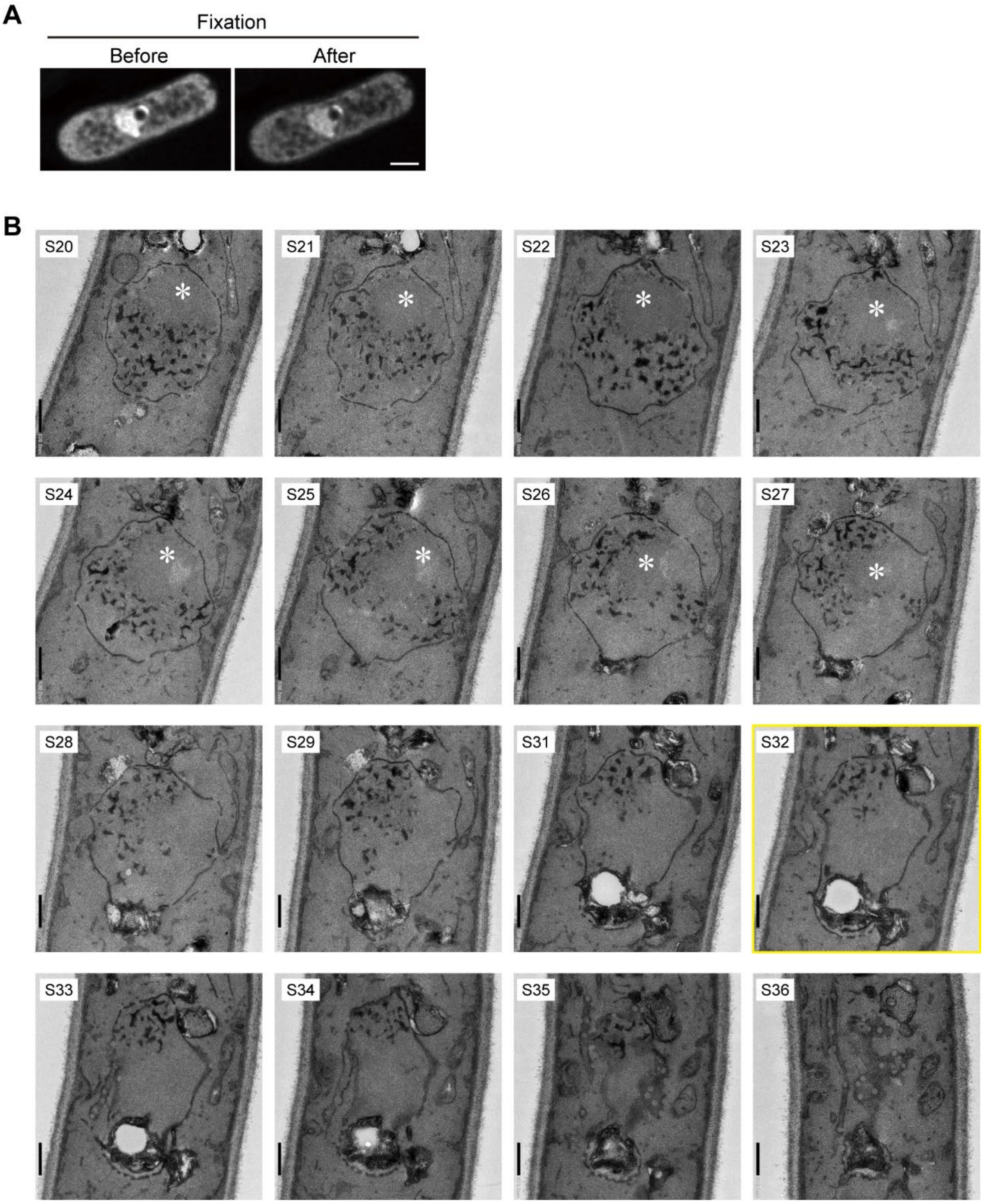

Supplementary Fig. S4. Serial section TEM images of the cell overexpressing IDR

A. Fixation did not alter the structure observed in living cells. Cells overexpressing IDR were observed in live (Before) and then fixed with PFA for 10 min. The same region observed in live imaging was observed again after fixation (After). The scale bar represents 2 μm.

B. Serial-section TEM images of cells overexpressing IDR are shown in Fig. 2H. The numbers on the upper left indicate the section numbers. S32 is shown in Fig. 2H. Asterisk at S20-S27 indicates the nucleolus. The scale bars represent 500 nm.

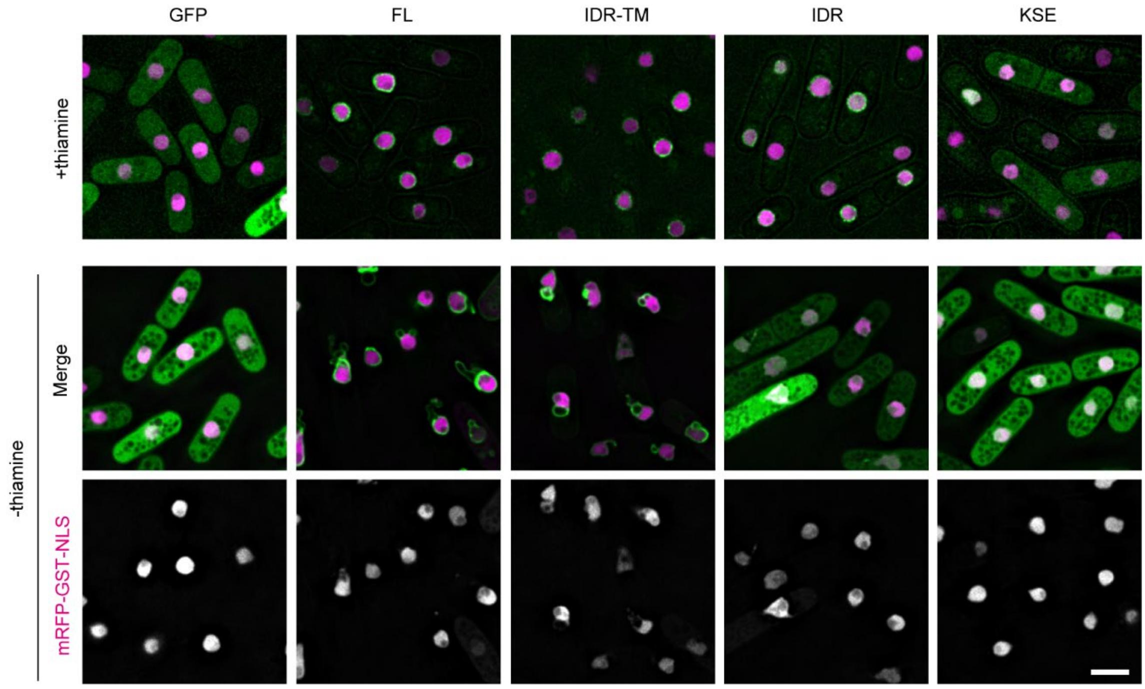

Supplementary Fig. S5. Overexpression of Bqt4 fragments did not disturb the NE integrity
Cells expressing Bqt4 fragments and mRFP-GST-NLS were cultured in the presence or absence of thiamine and observed using fluorescence microscopy. Scale bar represents 5 μm.

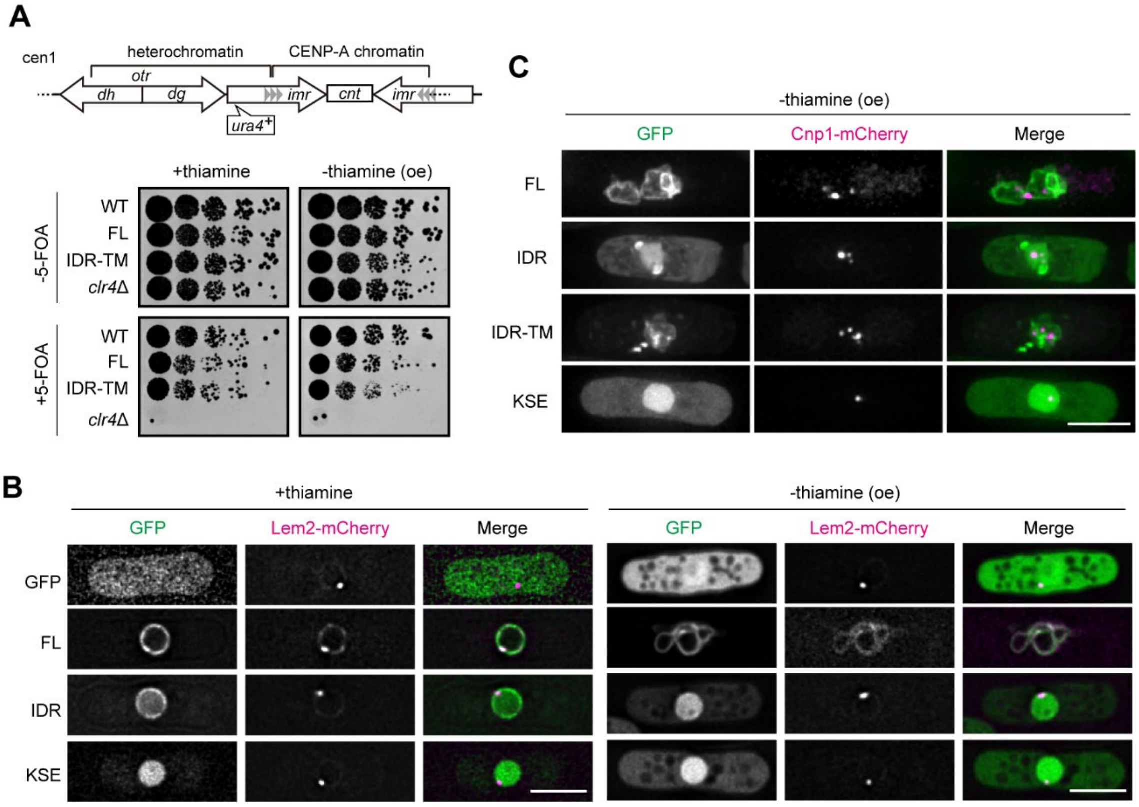

Supplementary Fig. S6. Lem2 did not involve centromeric defects caused by Bqt4 overexpression

A. Heterochromatin silencing assay in cells overexpressing GFP-FL or IDR-TM. The cells with increased expression of GFP-FL or -IDR-TM and *clr4*Δ cells were spotted on the EMMG containing 5-FOA with or without thiamine in five-fold serial dilutions and then grown at 30°C for 4 days.

B. Subcellular localization of Lem2 in the cells overexpressing Bqt4 fragment. GFP-tagged Bqt4 fragments (green) indicated on the left were expressed in the cells expressing mCherry-tagged Lem2 (Lem2-mCherry, magenta). The cells were cultured in the EMMG medium in the presence or absence of thiamine ("+thiamine" and "-thiamine (oe)", respectively). Lem2-mCherry is accumulated at the centromere in the control cells disrupted for the authentic *bqt4*$^{+}$ gene as previously reported (Hirano et al., 2018). The scale bars represent 5 µm.

C. Distribution of centromeres in *lem2*Δ cells. *lem2*Δ cells expressing the GFP or GFP-Bqt4 fragments indicated on the left (green) and Cnp1-mCherry (magenta) were cultured in EMMG medium and observed using fluorescence microscopy. The scale bar represents 5 µm.

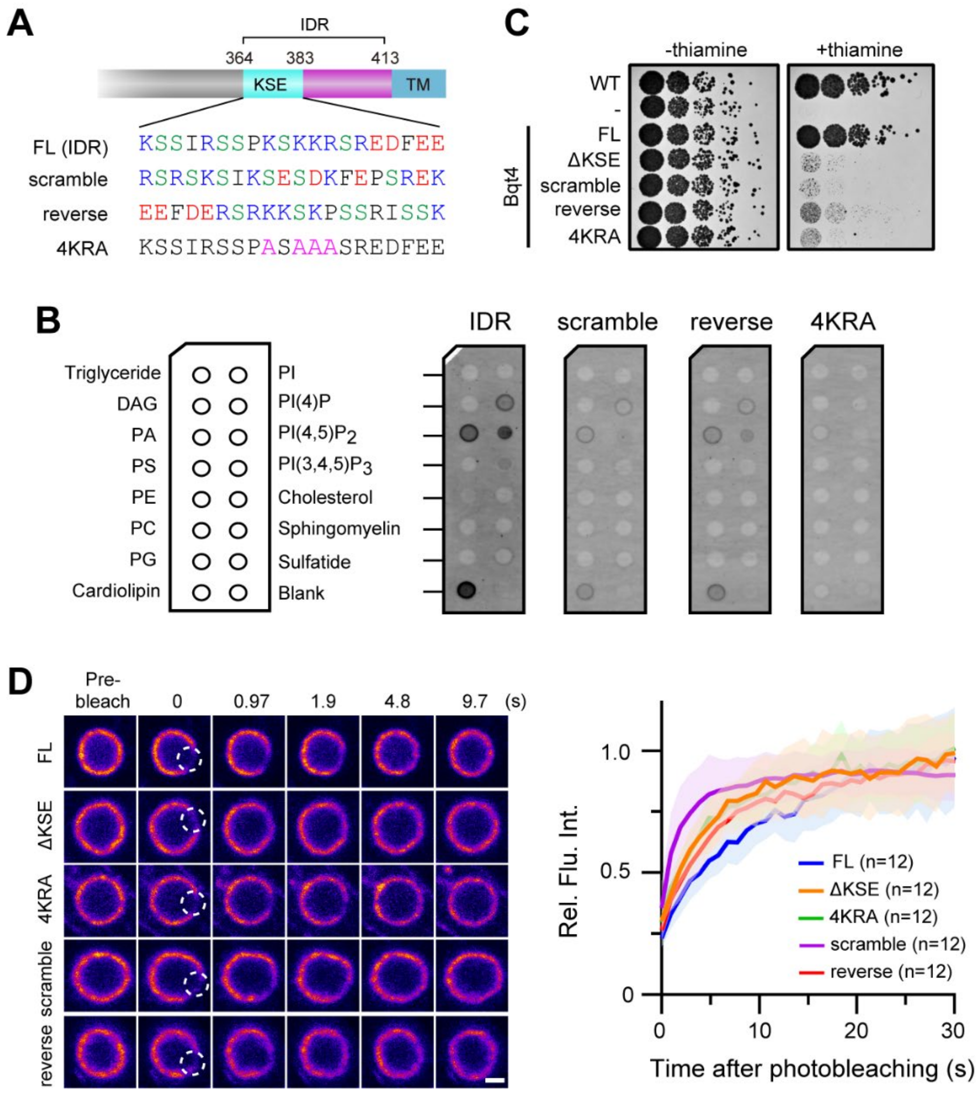

Supplementary Fig. S7. Disturbing the highly positive charge in the KSE region leads to the loss of the Bqt4 function

A. Bqt4 proteins used. We constructed Bqt4 mutants with scrambled or reversed amino acid sequences of the KSE region (scramble and reverse, respectively). The 4KRA is the same mutation shown in Fig. 4. These mutations were introduced in full-length Bqt4 (FL) and the IDR fragment (IDR) for spot and FRAP analyses and lipid-binding assay, respectively. Blue, red, green, and pink characters represent positive, negative, polar, and mutated amino acids.

B. Lipid binding assay. The schematic illustration on the left shows lipids spotted on the membrane lipid strip. His-GFP-tagged IDR, scramble, reverse, or 4KRA was incubated with the membrane and the bound protein was detected using fluorescence.

C. Spot assay of the mutants in the KSE region. The cells expressing mutants shown on the left were spotted and the growth was observed as described in Fig. 1B.

D. FRAP analysis of GFP-tagged Bqt4 fragments (FL, ΔKSE, 4KRA, scramble, reverse, and 4KRA). The dashed white open circle region was bleached, and the recovery of fluorescence intensity after bleaching in the circle was measured. Solid lines in the FRAP curve indicate the mean value. The areas on both sides of the solid lines indicate standard deviation. The scale bar represents 1 μm.

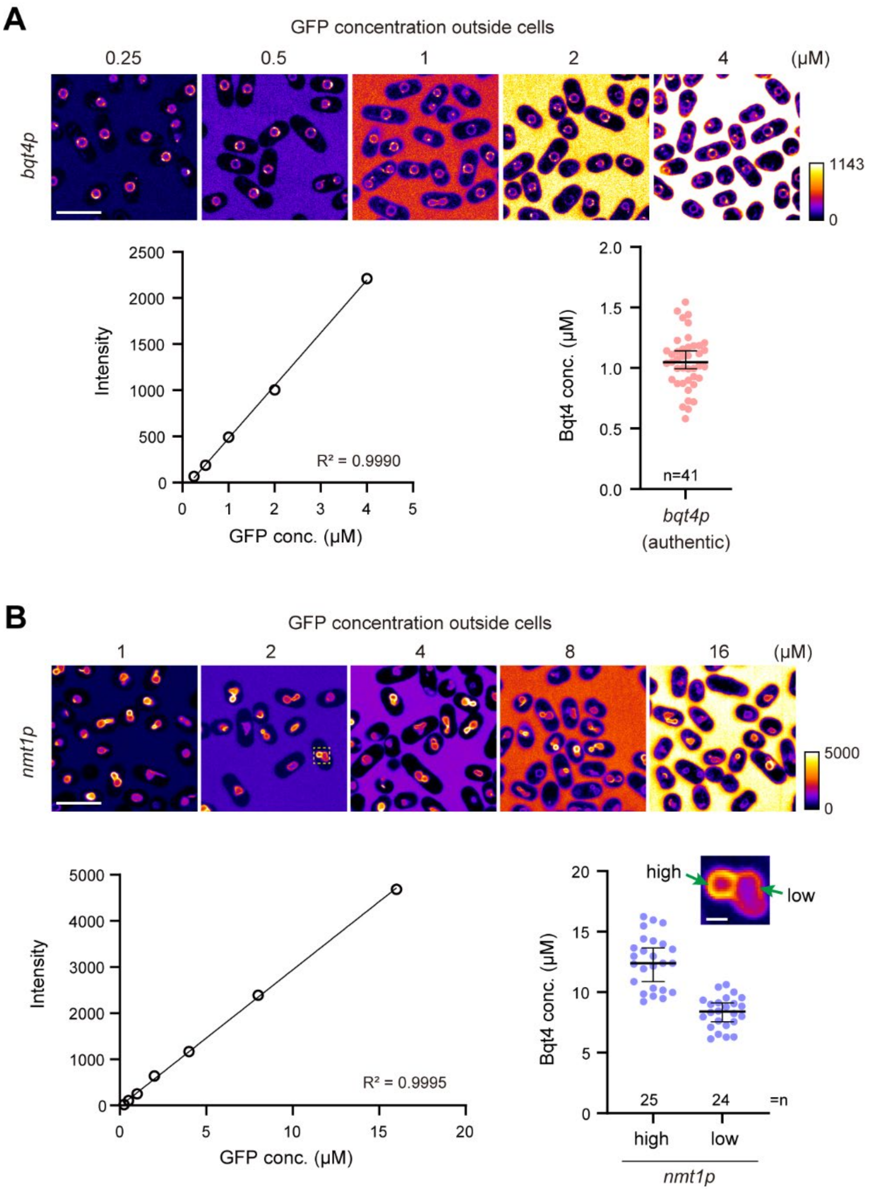

Supplementary Fig. S8. Bqt4 concentration in the NE

Cells expressing GFP-Bqt4 under authentic (A) or *nmt1* (B) promoters were mixed with purified GFP solutions of increasing concentrations indicated on the top and observed using fluorescence microscopy (top panels). A standard curve was constructed by quantifying the intensities outside of the cells (bottom left). Bqt4 concentrations in the NE were determined using a standard curve (bottom right). Since the Bqt4 concentration in the NE expressed under the *nmt1* promoter was not uniform, we categorized it into high- and low-concentration regions (inset of (B)). The horizontal line and whiskers in the plots denote median and 95% confidence intervals. The scale bars represent 10 μm for the top row images in (A) and (B), and 1 μm for the inset of (B).

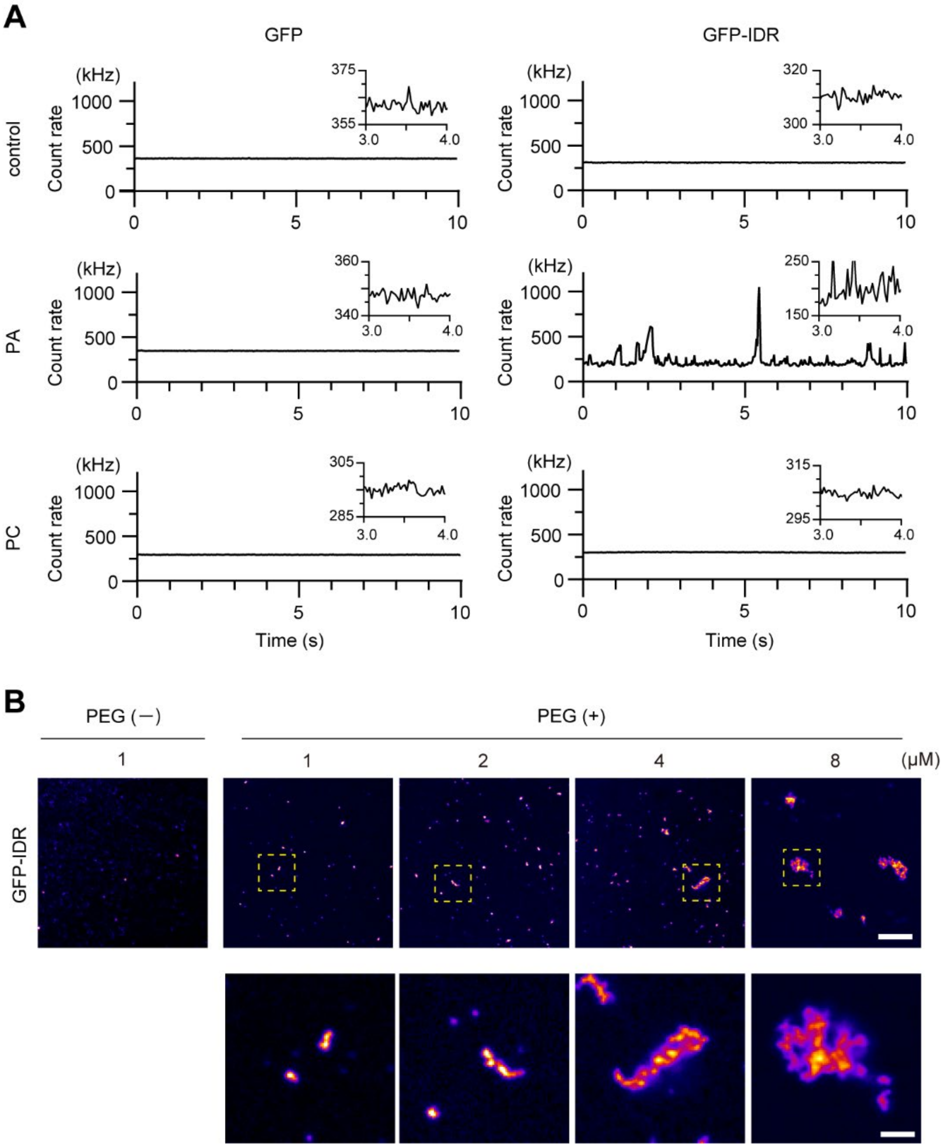

Supplementary Fig. S9. Molecular clustering of GFP-IDR

A. Fluorescence intensity fluctuation during FCS measurements. GFP (left) and GFP-IDR (right) were mixed with 100 nmol PA or PC, and then the mixture was analyzed using FCS. Inlets are enlarged images of the part of the count rate.

B. Concentration dependency of the aggregate formation of GFP-IDR. Increasing concentrations of GFP-IDR indicated on the top were incubated in a crowding solution in the presence or absence of 5% PEG ("PEG(+)" and "PEG(-)"). The formed aggregates were observed using a fluorescence microscope. The yellow dashed square regions are enlarged at the bottom of the figure. The scale bars in the top and bottom panels indicate 10 and 2 μm, respectively.

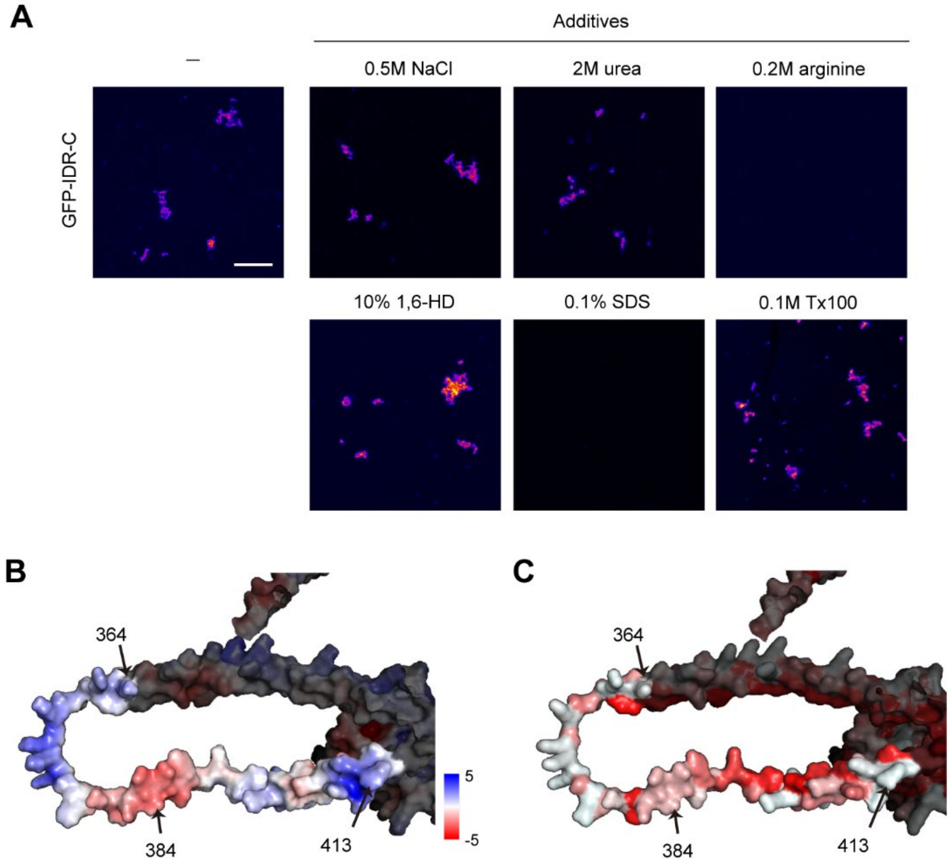

Supplementary Fig. S10. Effect of additives on GFP-IDR-C aggregate

A. Eight micromolar of GFP-IDR-C was incubated in a crowding solution supplied with additives indicated on the top, and the formed aggregates were observed using fluorescence microscopy. The scale bar represents 10 µm.

B. Electrostatics of IDR. The electrostatic condition of Bqt4 was calculated by the adaptive Poisson-Boltzmann solver plugin in PyMOL software and drawn on the predicted Bqt4 structure by Alphafold2 (O60158). The blue and red portions indicate amino acid residues with positive and negative charges, respectively. Bright residues correspond to 364-413 aa.

C. Hydrophobicity of IDR. Hydrophobicity around IDR is shown on the predicted Bqt4 structure as in (B). The red and white portions indicate hydrophobic and hydrophilic regions, respectively.

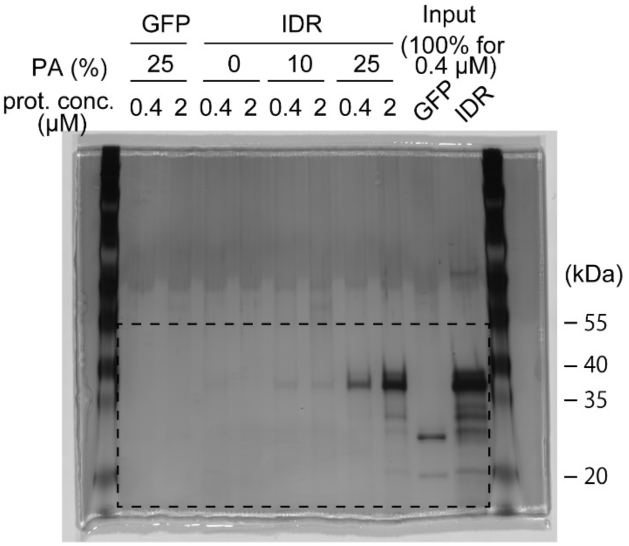

Supplementary Fig. S11. Uncropped gel image
The dashed square region is shown in Figure 4B.

Legends to Supplementary Movie

Supplementary Movie 1. Detachment and declustering of centromeres from the NE by Bqt4 overexpression

GFP-tagged IDR-TM (green) was expressed under the control of the *nmt1* promoter in cells expressing Cnp1-mCherry (magenta) as a centromere marker. The cells were pre-cultured in EMMG+1 μM YAM2 for overnight, washed, and then observed using fluorescence microscopy. The bright-field image is shown on the right. The elapsed time is denoted as h:min:s. The scale bar indicates 10 μm.

Supplementary Movie 2. Chromosome segregation defect by Bqt4 overexpression

GFP-tagged IDR-TM (green) was expressed under control of the *nmt1* promoter in cells expressing Atb2-mCherry (magenta). The cells were pre-cultured in EMMG+1 μM YAM2 overnight, washed, and then observed using fluorescence microscopy. The elapsed time is denoted as h:min:s. Scale bar: 5 μm.
